# Surviving extreme polar winters by desiccation: clues from Arctic springtail (*Onychiurus arcticus*) EST libraries

**DOI:** 10.1186/1471-2164-8-475

**Published:** 2007-12-21

**Authors:** Melody S Clark, Michael AS Thorne, Jelena Purać, Gordana Grubor-Lajšić, Michael Kube, Richard Reinhardt, M Roger Worland

**Affiliations:** 1 British Antarctic Survey, Natural Environment Research Council, High Cross, Madingley Road, Cambridge, CB3 0ET, UK; 2 University of Novi-Sad, Faculty of Sciences, Trg Dositeja Obradovića 3, 21000 Novi Sad, Republic of Serbia; 3 Max-Planck-Institut für Molekulare Genetik (MPI), Ihnestraße 63/73, 14195 Berlin, Germany

## Abstract

**Background:**

Ice, snow and temperatures of -14°C are conditions which most animals would find difficult, if not impossible, to survive in. However this exactly describes the Arctic winter, and the Arctic springtail *Onychiurus arcticus *regularly survives these extreme conditions and re-emerges in the spring. It is able to do this by reducing the amount of water in its body to almost zero: a process that is called "protective dehydration". The aim of this project was to generate clones and sequence data in the form of ESTs to provide a platform for the future molecular characterisation of the processes involved in protective dehydration.

**Results:**

Five normalised libraries were produced from both desiccating and rehydrating populations of *O. arcticus *from stages that had previously been defined as potentially informative for molecular analyses. A total of 16,379 EST clones were generated and analysed using Blast and GO annotation. 40% of the clones produced significant matches against the Swissprot and trembl databases and these were further analysed using GO annotation. Extraction and analysis of GO annotations proved an extremely effective method for identifying generic processes associated with biochemical pathways, proving more efficient than solely analysing Blast data output. A number of genes were identified, which have previously been shown to be involved in water transport and desiccation such as members of the aquaporin family. Identification of these clones in specific libraries associated with desiccation validates the computational analysis by library rather than producing a global overview of all libraries combined.

**Conclusion:**

This paper describes for the first time EST data from the arctic springtail (*O. arcticus*). This significantly enhances the number of Collembolan ESTs in the public databases, providing useful comparative data within this phylum. The use of GO annotation for analysis has facilitated the identification of a wide variety of ESTs associated with a number of different biochemical pathways involved in the dehydration and recovery process in *O. arcticus*.

## Background

The mechanisms by which organisms survive extreme low temperatures are not only of interest to ecologists, but also to a number of applied medical fields [1]. In this respect, one of the most amenable and studied groups of organisms is the Collembola (arthropods or springtails) where the physiological processes behind such survival are well documented [2,3]. They possess three main strategies to survive the cold: freeze tolerance, freeze avoidance or protective dehydration [3-5]. Whilst most springtails use freeze avoidance, it is the latter strategy of protective dehydration in the Arctic springtail *Onychiurus arcticus *(Tullberg 1876) which is the subject of this study [5,6]. In protective dehydration loss of water occurs across a diffusion gradient between the animal's super-cooled body fluids and ice in its surroundings, such that freezing point depression always exceeds the environmental temperature experienced, and eventually the animals lose sufficient water to ensure that a freezing event cannot occur [5,6]; the animals desiccate. *O. arcticus *is widely distributed throughout the northern parts of the Palaearctic region [7-10] and is found in moist habitats; mainly in mosses and under large stones in the coastal areas of Svalbard, particularly on glacial outwash fans and under bird cliffs [11]. Studies have shown that *O. arcticus*, exposed to sub-zero temperatures and low water vapor pressure induces extensive dehydration through a highly permeable cuticle [5,12,13]. This is combined with the rapid synthesis and accumulation of the membrane/protein cryoprotectant trehalose from glycogen [13-15].

Whilst there are a number of physiological and ecological studies on this organism (detailed above), there have been no molecular analyses to date (only *O. groenlandicus *has been bar-coded: AY665335, AY6653316, AY665323). This situation is not unusual, as the number of organisms where there is even moderate amounts of sequence data are severely limited. However, genomics is being increasingly applied to the study of non-model organisms and ESTs are generally viewed as the most efficient and cost effective strategy for the identification of genes and generating a first pass scan of a genome [16,17].

As part of a larger project examining over-wintering strategies in polar arthropods, we have generated 16,379 ESTs for *O. arcticus *from 5 cDNA libraries of animals in different desiccation states. In this article we present the analysis of these library data. This represents the first sequence data for this organism and significantly increases the number of Collembolan ESTs in the databases from the previous total of 8,686 produced from the springtail *Folsomia candida *[18].

## Results and Discussion

### CDNA libraries construction and characterisation

Five libraries were produced from both desiccating and re-hydrating populations of *O. arcticus *from stages that had previously been defined as potentially informative for molecular analyses [13].

• *Library C*: Controls

• *Library D1*: Desiccating: animals at -2°C. This is the critical temperature at which trehalose is significantly up-regulated at the expense of stores of glycogen.

• *Library D2*: Fully desiccated animals.

• *Library R1*: Animals that had been recovering for 8 hours.

• *Library R2*: Fully recovered animals.

For more detail, see methods. A total of 16,552 clones were sequenced from the 5' direction. This was reduced slightly to 16,379 clones after quality checks, each of which had a minimum transcript size of 150 bp and an average of 550 bp. Each library proportionally contributed between 17 and 23% of the total EST dataset (Table 1). The libraries were normalised to optimise the chances of obtaining rare transcripts. Indeed, both gene diversity and discovery ratios were high in all libraries, with a minimum diversity score of 79% (61% for all libraries) and even the largest clusters comprising only approximately 0.4% of any one library (Table 1). When sequence similarity searches were run on the processed sequences, on average 40% produced significant matches (expect score in excess of 1e-10 and therefore can be regarded as putative known genes) against the sequence databases. This level of gene identification is not surprising and recent EST data generated from the springtail *Folsomia candida *(Timmermans et al, 2007) produced similar levels of BLAST identity. This is largely because there are a limited number of insects that have been sequenced. The two main insects in the ensembl database [19] are *Drosophila melanogaster *and *Anopheles gambiae*, which are only related to *O. arcticus *at the Super class level (Hexopoda). Of the two, *A. gambiae *has most recently been sequenced and the VectorBase genome build from June 2007 (AgamP3) produced only 10% known genes from a 10 fold genome sequence coverage of 278 Mb. Similarly recent EST data (in excess of 150,000 ESTs) from the silkworm (*Bombyx mori*) only provides confirmation of 15% of the predicted genes from the WGS project [20-22].

**Table 1:** 

**Library**		**C**	**D1**	**D2**	**R1**	**R2**
		
**Condition**	**All**	**Control**	**Desiccating -2°C**	**Desiccated -14°C**	**Recovering 8 hr**	**Recovered 24 hr**
# Reads*	16379	2865	3414	3449	3852	2799
**Average length**	557	564	548	555	559	560
**# singletons**	7174	2205	2432	2276	2488	2186
**# clusters (> 1 read)**	2795	269	409	463	542	265
**# putative transcripts**	9969	2474	2841	2739	3030	2451
**Average cluster size**	3.29	2.45	2.40	2.53	2.52	2.31
**Largest cluster (# reads)**	30	14	12	10	8	6
**# clusters with 2 ESTs**	1469	195	302	318	354	205
**# clusters with 3 ESTs**	549	48	72	86	133	42
**# clusters with 4–5 ESTs**	464	21	30	48	45	16
**# clusters with 6–10 ESTs**	264	4	4	11	10	2
**# clusters with > 10 ESTs**	49	1	1	0	0	0
**Gene Discovery**	0.44	0.75	0.71	0.64	0.65	0.78
**Gene Diversity**	0.61	0.85	0.83	0.79	0.79	0.88
#(%) with significant **Swissprot hits	3429 (34%)	790 (32%)	1085 (38%)	1014 (37%)	1130 (37%)	814 (33%)
#(%) with significant **trembl hits	3970 (40%)	916 (37%)	1145 (40%)	1196 (44%)	1282 (42%)	934 (38%)
**#(%) with no hits**	5966 (60%)	1548 (63%)	1602 (56%)	1535 (56%)	1726 (57%)	1507 (61%)

General statistics for all libraries individually and combined. * Reads that are submittable and >150 bp. ** Threshold for Blastx significance = 1e-10. Gene discovery is defined as the number of different "genes" each library contributed, divided by library size. Gene Diversity is defined as the number of singletons in each library divided by library size [59].

**Figure 1:**
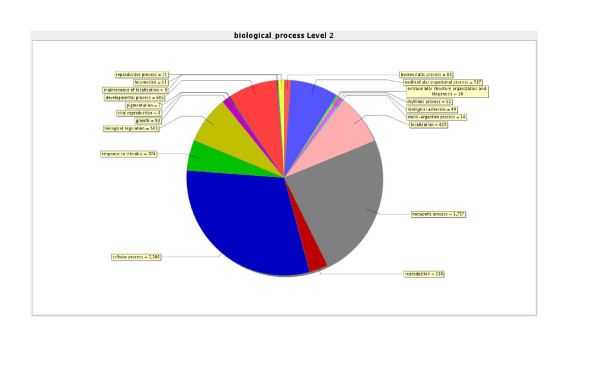
Example of GO molecular function (level 2) characterisation of the libraries, the data from all libraries combined is shown.

The sequences from each library were further processed using Blast2GO [23], but showed very similar compositions when defined in terms of their molecular function (GO annotation level 2). An example of this output is shown for all libraries in Figure 1, with the majority of clones having either catalytic or binding activities. Although the libraries were normalised, GOSSIP [24] was used to perform statistical analyses using pair-wise comparisons of each library with the control library to identify any potential enrichment for particular genes or gene functions. No enrichment was found using the corrected p-values of the False Discovery Rate, although there were a number of significant single test p-values. For example GO016860: intramolecular oxidoreductase activity and GO008237: metalloexopeptidase activity were both elevated in the desiccated library compared to the control with single test p-values of 0.05, these were not significant using the False Discovery Rate. The problem with such pair wise comparisons is that even though the comparative library was always the control animals, the GO categories listed for each pair wise comparison varied considerably and it was not possible using this technique to make global statements of certain molecular functions being statistically enhanced in one library compared to the others. These data do indicate that the normalisation procedure in the library production process was relatively effective.

### GO annotation

The decision was made to analyse the libraries using GO annotation rather than keyword extraction from Blast comment lines, as GO categories are more generic than gene names. This facilitates the comprehensive identification of gene sets associated with biochemical pathways without in-depth knowledge of individual pathway components and also where potentially multiple pathways exist for a particular process (cf. trehalose [25]). In the following discussion PGO refers to a Biological Process GO annotation; FGO is Molecular Function and CGO is Cellular component.

Initially the focus of the searches concentrated on general processes such as response to water, water deprivation, abiotic stimulus, desiccation and Qdrought recovery. Disappointingly only 11 clones were identified from the five libraries, but even so, these did include some clones of further interest. PGO:0009414, response to water deprivation identified a putative aquaporin (the same clones were also identified under a specific GO search for aquaporin) and PGO:0009415, response to water, produced ESTs with matches to dehydrin which is induced in response to water stress in plants, the latter of which would not have been identified using extraction of Blast keyword data. Given the relative lack of success of the generic searches, specific genes and pathways were targeted. Of the genes present in the libraries, there was particular interest in identifying the following genes:

• The trehalose and glycogen pathways (as trehalose is produced from glycogen during the desiccation process) [25].

• Aquaporins: 6 transmembrane domain proteins involved in water transport [26,27].

• Genes involved in cell protection, such as antioxidants

• Genes involved in moulting, as moulting has previously been implicated in lowering the supercooling point and survival ability of Antarctic springtails (*Cryptopygus antarcticus*) [28,29].

• LEA (Late Embryogeneis Abundant) proteins, which have been shown to be involved in desiccation in a number of organisms [30].

### Trehalose and glycogen pathways

Initially the trehalose pathway was screened only for the GO molecular functions directly associated with it, but this revealed very few matching clones, with only 3 clones for FGO:0003825 (alpha, alpha-trehalose-phosphate synthase (UDP-forming) activity, none for FGO: 0015574 (trehalose transmembrane transporter activity) and 3 for FGO:0004805 (trehalose-phosphate activity) (Table 2). Because of the redundancy in levels of GO annotation, all six clones identified by the molecular processes matched the same gene: trehalose-6-phosphate synthase (probability and expect scores in excess of 493, 1.6 e^-44^). A similar situation occurred when identifying genes involved in the glycogen pathway, in that a number of clones were identified (137 in total), but many were to the same gene: glycogen phosphorylase which breaks up glycogen into glucose subunits.

**Table 2:** 

	**C**	**D1**	**D2**	**R1**	**R2**
**TREHALASE**					
alpha, alpha-trehalase activity; FGO:0004555	0	2	1	1	1
trehalase activity; FGO:0015927	0	1	1	0	1
*Total*	*0*	*3*	*2*	*1*	*2*
					
**TREHALOSE**					
**alpha, alpha-trehalose-phosphate synthase (UDP-forming) activity; FGO:0003825**	0	1	1	1	0
alpha, alpha-trehalose-phosphate synthase complex (UDP-forming); CGO:0005946	0	0	0	1	0
trehalose biosynthetic process; PGO:0005992	1	1	2	2	1
trehalose catabolic process; PGO:0005993	0	1	1	0	1
**Trehalose metabolic process; PGO:0005991**	**1**	**3**	**10**	**2**	**3**
**Trehalose transmembrane transporter activity; FGO:0015574**	0	0	0	0	0
trehalose transport; PGO:0015771	0	0	0	0	0
**Trehalose-phosphatase activity; FGO:0004805**	0	1	1	1	0
*Total*	*2*	*7*	*15*	*7*	*5*

GO annotation matches according to library for both molecular function and biological process associated with the trehalose pathway. Highlighted names indicate molecular function and the highlighted row indicates the biological process clones that also investigated with Blast data.

Further analysis was carried out on the trehalose pathway by expanding the search to the biological process PGO:0005991 (trehalose metabolic process). This produced 19 matches in total. In-depth analysis of these revealed matches to trehalose-6-phosphate synthase, trehalase precursor and a number of different protein kinases (Table 3). These included a serine-threonine protein kinase from the yellow fever mosquito, which in humans is involved in phosphorylation and protein kinase A cAMP-dependant catalytic subunit from the same organism. Phosphorylation is an integral process in trehalose biosynthesis and degradation, so protein kinases are certainly involved. The question is, which ones? The GO annotations, whilst largely based on mammalian data, provide immediate candidates for further analysis, which would not have been identified using screening of Blast outputs using specific keywords for the trehalose pathway enzymes. It is interesting to note that libraries produced from control animals did not contain any matches to trehalase activity, which is associated with the breakdown of trehalose. However, this is to be expected as biochemical studies indicate that trehalose is largely absent in control animals and that it is only produced in response to the desiccation process [13]. Also that the libraries prepared from the desiccated animals contained the highest number of potential matches to trehalose synthesis GO annotations, which is where it is expected that most trehalose would be produced.

**Table 3:** 

**UniRef ID**	**GENE**	**ORGANISM**	**GO**	**P**	**EXPECT**	**CLONES**
A4GHG0	Trehalose 6 phosphate synthase	*Spodoptera exigua *(beet armyworm)	FGO:0003825 FGO:0004805 PGO:0005991	796	2.6e-77	sb_006_04P17
			FGO:0003825	493	1.6e-44	sb_007_01G20
			FGO:0004805	808	1.3e-78	sb_009_07N11
Q24096	LATS tumour suppressor	*Drosophila melanogaster*	PGO:0005991	500	7.5e-45	sb_005_07N02
				774	7.8e-75	sb_006_04E02
Q16U52	Serine/threonine protein kinase 38	*Aedes aegypti *(yellow fever mosquito)		1093	8.5e-109	CL107 (LIB006)
Q1HQW4	Protein kinase A camp dependant catalytic subunit	*Aedes aegypti*		589	2e-55	CL151 (LIB006)
				1259	2.2e-126	CL156 (LIB006)
				649	9.4e-62	sb_006_07I02
				693	2.1e-66	CL543 (LIB007)
				596	3.8e-56	sb_008_06E21
Q9BLC8	Trehalase precursor	*Artemia sanfranciscana *(brine shrimp)		459	3.0e-41	sb_006_01B14
				440	4.2e-39	sb_008_02F17
				457	5.1e-41	sb_009_04C10
Q9GQB3	P70 ribosomal protein S6 kinase	*Artemia sanfranciscana*		533	1.7e-49	sb_006_01P13
Q194S5	Putative protein kinase DC2	*Drosophila Melanogaster*		388	4.3e-34	sb_005_C18
Q96GD4	Similar to serine/threonine protein kinase 6 (Aurora family kinase 1)	*Apis mellifera *European honey bee		581	1.5e-54	sb_006_07A19
				583	9.1e-55	sb_007_01L21
Q17N56	Protein kinase C	*Aedes aegypti*		606	3.2e-57	sb_008_03M09
A5JNM1	CAMP dependant protein kinase C1	*Bombyx mori *(silk moth)		783	6.2e-76	sb_009_02D08
				796	2.5e-77	sb_009_06E07

Database matches for "genes" identified from GO annotations for both the metabolic function and biological processes associated with trehalose metabolism. Matches to the databases are shown with probability and expect values. Organism names are shown with the common name at first annotation.

### Aquaporins

These proteins are associated with water transport across membranes (Kruse et al, 2006) and have the GO annotation: FGO:0015250: water channel activity. Searches of all libraries revealed 7 clones in total (4 singletons and a cluster of 3) matching three potentially different aquaporin genes:

• Q0IG28: Aquaporin 1 from Aedes aegypti. Two clones from control and desiccating libraries (C and D1), matches in excess of P value = 177, expect value = 9.7e-12.

• Q9NHW7: Aquaporin AQPAe.a from Aedes aegypti. Two clones from desiccated and recovering libraries (D2 and R2), matches in excess of P value = 512, expect value = 3.2 e-47

• UniRef100 000051A00B: Predicted similar to Drip CG9023_PB isoform B Apis mellifera. 1 cluster of 3 genes from the desiccated library (D2) with a match of P value = 480, expect value = 7.7 e-44

**Figure 2:**
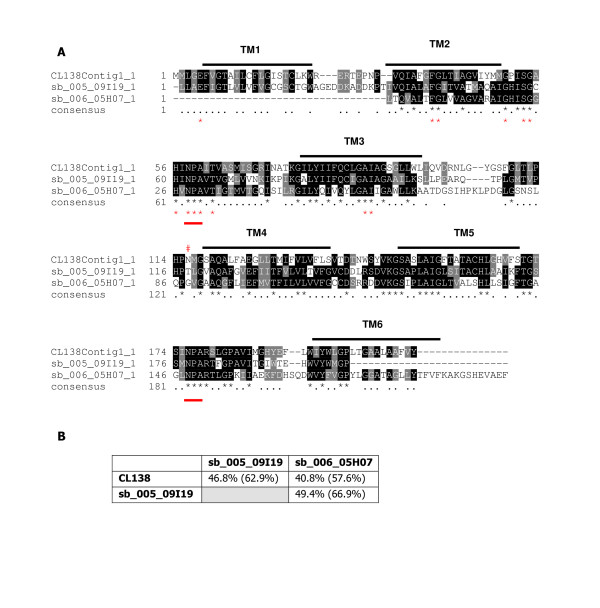
Alignment of the three putative aquaporin clones identified in the libraries. Transmembrane domains are marked above the sequence (TM6 is only partial in both CL138 and sb_ 005_09I19). Red lines denote the two conserved NPA motifs of the aquaporin family. Only CL138 has conserved the site for N-glycosylation [31]. Red asterisks below the consensus line identify 13/14 amino acids conserved throughout the mammalian aquaporin family, as outlined in previous protein fragment analyses [59]. **B) **Percentage amino acid identities between the different springtail aquaporin clones. Figures in brackets are the percentage amino acid similarities. Each clone was clipped to the same size when performing the calculations.

**Figure 3:**
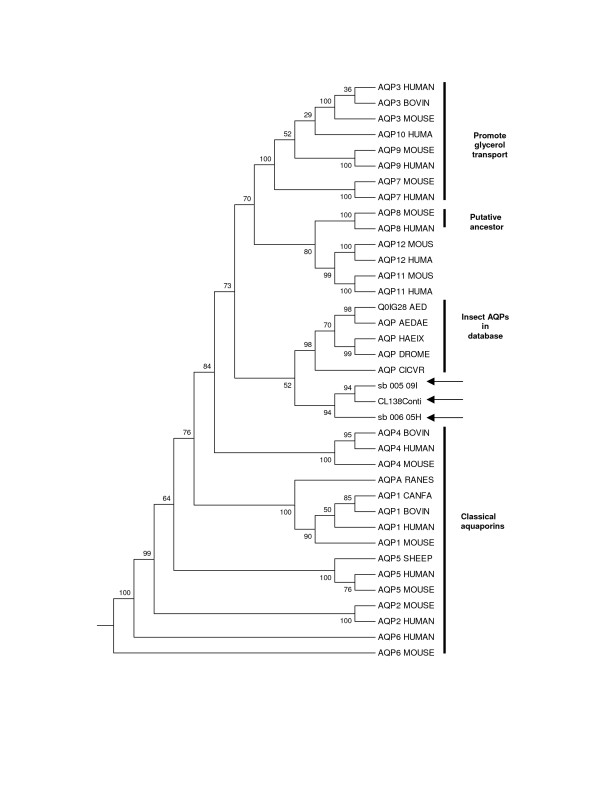
Maximum parsimony tree of 12 aquaporin genes and the three *O. arcticus *clones (sb_005_09I19, CL138 and sb_006_05H07) (arrowed). Accession numbers: Human AQPs 1-12A: P29972, P41181, Q92482, P55087, P55064, Q13520, O14520, O94778, O43315, Q96PS8, Q8NBQ7, Q8IXF9; Mouse AQPs 1-12A (10 is absent): Q02013, P56402, Q8R2N1, P55088, Q9WTY4, Q8C4A0, O54794, P56404, Q9JJJ3, Q8BHH1, Q8CHJ2. Bovin AQPs 1, 3, 4: P47865, Q08DE6, O77750. Dog (CANFA) AQP 1: Q9N2J4. Sheep AQP5: Q866S3. Frog AQP (RANES) (Rana esculenta): P50501. Insect AQPs: Q0IG28, Q9NHW7: AEDAE: *Aedes aegypti *(yellow fever mosquito); Q23808: CICVR: *Cicadella viridis *(green leaf hopper); Q9V5Z7: DROME (*Drosophila melanogaster*) (fruit fly); Q25074: HAEIX (*Haematobia irritans exigua*) (buffalo fly).

Translation of the individual clones and alignments confirmed that three different aquaporin genes had been cloned (Figure 2A) with a maximum of 49.4% amino acid identity between clones when compared over identical lengths (Figure 2B). The clones were all around 200 amino acids in length, which is approximately 80% of the expected length of an aquaporin gene. All clones included 5 complete transmembrane (TM) domains with TM6 present in sb_006_05H07 and partial in the two other clones. All three contained the classical footprint of aquaporins: two NPA motifs, cysteine 181 was not conserved and only CL138 had the consensus site for N-linked glycosylation. The consensus site for phosphorylation by protein kinase C was not present in any of the clones [31]. Homology between the clones was relatively low at a maximum of 49.4%, but not surprising as homology within aquaporins is generally low. For example the maximum homology between the latest aquaporin identified in mouse (AQP12) with the other mouse AQPs is 38.9% [32]. When the 328 bp sequence from exon 1 of the gene for aquaporin-2 was compared in 12 mammalian species, only 14 out of 109 amino acids were conserved throughout the mammalian aquaporin family, of these 13 were conserved in the springtail AQPs (Figure 2A). Identity of the springtail clones was confirmed by phylogenetic analysis (Figure 3). All three clones clustered with the other insect aquaporins extracted from the database. Interestingly all insect clones clustered more strongly with the aquaglyceroporins, AQP8, the putative ancestral molecule [27] and AQP 11 and 12. Relatively little is known about the latter two genes, but the human AQP12 is hypothesised to play a novel intracellular role in digestive enzyme secretion [32]. Limited functional analysis has been carried out on the insect aquaporins, but evidence points toward them being designated as classical water channel molecules [33,34]. *Cicadella viridis *AQPcic has been shown to increase osmotic membrane water permeability when expressed in *Xenopus *oocytes and the *Aedes aegypti *AQP (Accession number: Q9NHW7) gene was localised to tracheolar cells associated with malpigian tubules and therefore it was hypothesised that this protein played a role in the removal of tracheolar fluid during respiration.

### Cell protection

A general search for the level 2 GO annotation FGO:0016209 revealed 42 potential clones with antioxidant activity from all 5 libraries. However because of the complexity of the GO annotation network, further searches were carried out for level 3 molecular functions and biological processes under specific functions: hydrogen peroxide, melanin, catalase, superoxide dismutase, glutathione, glutathione transferase and glutathione reductase (Table 4). These identified a greater number of clones (387), many of which were duplicated between the libraries (data not shown). It is interesting to note that the highest number of antioxidant clones were present in both the desiccating and desiccated libraries (D1 and D2). From the percentages of clones present in each library for each antioxidant, the major components would appear to be glutathione, catalase and hydrogen peroxidase, with melanin and superoxide dismutase playing minor roles.

**Table 4:** 

	**LIB C**	**LIB D1**	**LIB D2**	**LIB R1**	**LIB R2**
**HYDROGEN PEROXIDE**					
Number of clones	18	24	17	16	11
%	27	22	20	20	23
**MELANIN**					
Number of clones	2	5	5	3	1
%	3	6	6	4	2
**CATALASE**					
Number of clones	14	19	18	19	9
%	21	17	21	24	19
**SUPEROXIDE DISMUTASE**					
Number of clones	3	9	7	6	4
%	5	8	8	8	8
**GLUTATHIONE TRANSFERASE**					
Number of clones	8	16	10	12	10
%	12	15	12	15	21
**GLUTATHIONE REDUCTASE**					
Number of clones	6	5	5	4	0
%	9	5	6	5	0
**GLUTATHIONE**					
Number of clones	16	32	22	18	13
%	24	29	26	23	27
					
**TOTAL**	**67**	**110**	**84**	**78**	**48**

GO annotation matches according to library for both molecular function and biological process associated with antioxidant activity. Total number of clones for each antioxidant is shown along with the relative percentage that they comprise of the total antioxidant clones identified in each library.

### Moulting

Processes involved in the survival of insects at low temperatures include the removal or deactivation of ice nucleating agents, accumulation of cryoprotectants and thermal hysteresis proteins [35-38]. Moulting has recently been shown to be associated with reduction of the supercooling point (SCP) and hence cryoprotection in Antarctic springtails (*Crypotpygus antarcticus*) [28,29]. This might be expected to depress the SCP, because in Collembola the mid-gut and its entire contents are shed during moulting [39] resulting in the expulsion of potential ice nucleators in the animal gut. The physiology and timing of moulting in *O. arcticu*s has not been documented to date, although with a rigid exoskeleton they clearly moult regularly in order to grow. So genes and pathways involved in moulting were also investigated using GO annotations (data not shown). A number of relevant genes were identified for both juvenile hormone (a pleiotropic hormone, which in concert with ecdysteroids orchestrates moulting and metamorphosis and may be involved in reproduction in some species) and members of the ecdysone pathway. The latter included the ecdysone receptor and the protein ultraspiracle (XR2C) chorion factor. In addition to a considerable number of putative transcription factors and chromatin remodelling subunits were identified. It was interesting to note that more matches were found in the actively desiccating (D1) and recovering animals (LIBs R1 and R2) (10, 24 and 14 respectively, total = 48) compared to only 8 in the control and 5 in the desiccated populations. Thus indicating that moulting may either play a role in the desiccation process, or potentially is triggered in some animals by the cellular stress involved in desiccation and recovery. We are currently investigating the role of moulting in desiccation of *O. arcticus *using a biochemical test for 20-hydroxy ecdysone and are also actively collecting animals that have either just moulted or are in the process of moulting for more detailed molecular analysis.

### Lea proteins

**Figure 4:**
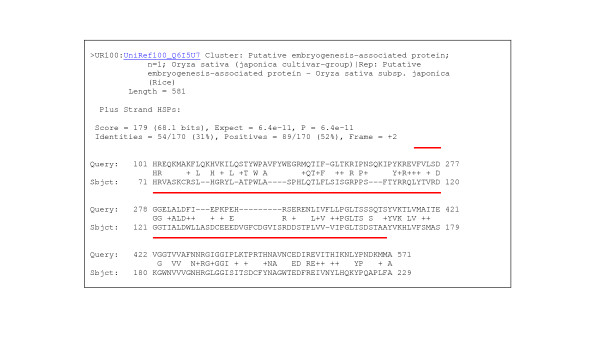
BLAST match to a putative LEA from rice. Line above the alignment denotes homology to an abhydrolase domain.

Of the genes and pathways under investigation, only LEA proteins were without GO annotations. So a search was made of the ESTs using Blast annotations. Identification of LEAs is problematical as these proteins are not highly conserved [30]. Comparison of LEA proteins between different species reveals only 53.5% identity between two cereal crops (Q42376: LEA3, maize and Q03968: LEA3, wheat), which reduces dramatically to 27.2% between different phyla such as chick pea and nematode (O49816 and Q95V77 respectively). Even the 11 amino acid repeat unit, which is a feature of LEAs shows little conservation with only 1 or 2 identical amino acids between chick pea and the nematode *Aphelenus avenae*. Searches of the *O. arcticus *BLAST annotations revealed a cluster of two clones present in Library D2 (desiccated animals) and a single clone in Library D1 (desiccating animals). This is exactly where such genes would be expected if they were involved in the desiccation process. Translation of all three clones and alignments indicated that only one gene was present. The primary BLAST match results for this clone against the databases were to Q1DH19, a putative uncharacterised protein from the yellow fever mosquito. After a number of subsequent matches to uncharacterised insect proteins, there were then matches to abhydrolase genes and LEA proteins (*Oryza sativa*) with probability and expect scores for the latter of 179 and 6.4e^-11 ^(Figure 4). At the amino acid level, identities were low with the best match to the uncharacterised protein showing 45% identity and the LEA protein, 31%.

Database searches of LEA proteins can produce matches to abhydrolase genes (as happened with this clone), due to the presence of an abhydrolase domain (denoted in Figure 4). Abhydrolases are a largely uncharacterised protein family, but members of this family do contain domains with hydrolase activity and therefore could potentially be involved in desiccation biochemistry. Phylogenetic analysis of the *O. arcticus *translated gene fragment suggests that this fragment most closely matches the ABHD_A (or ABHD_10) abhydrolase domain (data not shown). The question remains, is this clone sb_009_02E03 an LEA or an abhydrolase? This is virtually impossible to answer with short sequence fragments and the answer may become apparent with the full-length sequence, the cloning of which by RACE PCR is now underway. Also Western blotting studies are being carried out on *O. arcticus *using heterologous probing of a LEA antibody to identify such proteins via an alternative route.

### Differences between the libraries

All libraries were normalised during the construction process and therefore in-depth expression analysis was difficult. The primary aim of this EST production was to produce clones for a microarray and therefore normalisation was considered the best option for maximising gene discovery. However, lack or gain of gene expression could be inferred by presence or absence in some libraries. Certainly some GO annotations revealed similar distributions and levels of function across all libraries. But where very specific enzyme reactions or genes were identified some differences between libraries could be discerned using a plus/minus system cf. aquaporins and putative LEAs, as discussed above (Table 5). The identification of candidate genes for involvement in the desiccation process and some generalised differential expression between libraries has to be further verified using more comprehensive laboratory analyses such as RACE PCR to obtain full-length clones, microarrays and Q-PCR.

**Table 5:** 

	**LIBRARY**				
	
**GENE**	**CONTROL**	**DESICCATING (-2°C)**	**DESICCATED (-14°C)**	**RECOVERING (8hrs)**	**RECOVERED (24 hrs)**
	
	**LIB C**	**LIB D1**	**LIB D2**	**LIB R1**	**LIB R2**
Putative LEA protein		+	+		
*Aquaporins*					
• Aquaporin 1	+		+		
• Aquaporin AQPAE.a			+		+
• Aquaporin CG9023			+		
Abhydrolase		+	+		
Trehalase activity		+	+	+	+

Differences between the libraries for specific clones.

## Conclusion

This paper describes, for the first time, EST data from the arctic springtail (*O. arcticus*), significantly enhancing the number of Collembolan ESTs in the public databases. 40% of the clones produced significant matches against Swissprot and trembl and these ESTs were further analysed using GO annotations. This facilitated the identification of genes involved in biochemical pathways of interest, such as trehalose biosynthesis and moulting. The GO annotations produced a greater range of potential "genes" for further investigation and was more effective at identifying genes in a particular pathway than could have been identified using extraction of data from Blast. Candidate genes involved in the desiccation process were identified including three members of the aquaporin family and a putative LEA protein. These genes are under further investigation. The GO annotations identified in this publication will be used to automatically extract EST clone ids from in-house produced insect libraries to target further investigations into over-wintering survival of insects in extreme environments. This will include construction of customised microarrays.

## Methods

### Sample collection and preparation

*Onychiurus arcticus*, were collected under the bird cliffs at Stuphallet and Krykkefjellet on the Brøggerhalvøya, near Ny Ålesund, Spitsbergen, Svalbard, Norway (78°56'N, 11°53'E) and transported to the British Antarctic Survey (BAS), Cambridge, for analysis. Animals (mixture of both adult and juveniles) were cultured in ventilated plastic boxes containing moss, lichen and soil taken from field sites and fed on dried baker's yeast. Cultures were kept moist at +4°C.

Five groups of animals were prepared for library production:

• *Library C*: Controls: live animals which were kept in a +4°C cabinet

• *Library D1*: Desiccating: animals were cooled for two weeks from +2 to -2°C in culture pots containing a base of wet plaster of Paris/charcoal at a rate of 2°C per week. -2°C is the critical temperature at which trehalose is significantly up-regulated at the expense of stores of glycerol.

• *Library D2*: Fully Desiccated: animals were cooled from +2 to -14°C in culture pots containing a base of wet plaster of Paris/charcoal at a rate of 2°C per week.

• *Library R1*: Recovering: animals from the -14°C group were allowed to recover at +5°C with moisture for 8 hours.

• *Library R2*: Fully recovered: animals from the -14°C group were allowed to recover at +5°C moisture for 24 hours.

All groups of animals were rapidly frozen in liquid nitrogen and kept at -80°C until required.

### Library construction

Total RNA was extracted from frozen (-80°C) samples of *O. arcticus *using TRI Reagent (Sigma) according to manufacturer's instructions. cDNA was synthesized and normalized using a combination of the Trimmer-Direct kit (Evrogen, Moscow, Russia) and the SMART cDNA Library Construction kit (BD Biosciences Clontech, Palo-Alto, CA) according to the protocol from Evrogen [40]. Modifications to the protocol were made concerning the columns used for size selection and the cloning vector: to improve clone size selection Chroma spin 1000 were used instead of Chroma spin 400 (both BD Biosciences Clontech, Palo-Alto, CA) and pal32 (Evrogen, Moscow, Russia) was used for directional cloning with insertion between two SfiI sites (GGCCATTACGGCCGGG del(CATGTC) GGCCGCCTCGGCC. This procedure was chosen because of the low amount of starting material [41] and the normalisation process increased the efficiency of rare transcript discovery [42,43]. Plasmids were transferred via electroporation to *Escherichia coli *(strain DH10B, Invitrogen, Karlsruhe, Germany).

### cDNA Sequencing

Plasmids were isolated according to the method of [44] and 5'end sequenced using Dye Terminator Chemistry version 3.1 (ABI, Weiterstadt, Germany) and 3730XL ABI capillary sequencer systems (ABI, Weiterstadt, Germany).

### Sequence analysis and EST clustering

Sequence fasta files were processed using the script Trace2dbest [45], which incorporated the phred [46,47] and crossmatch (P. Green, unpublished) programmes. A minimum cut-off value of 150 bp was applied after quality control processing for sequence database searching and for generating the submission file for dbEST [48] (Accession numbers, dbEST: 49109381–49125759, Genbank: EW744731–EW761109). Tgicl [49] was used for clustering the fasta files, incorporating quality scores, for each of the five libraries, as well as for all the libraries together. The clusters were database searched using Blastx [50] against the Uniprot/Swissprot and Uniprot/Trembl databases [51] (at 12/06/2007), with matches annotated for all scores with an expect score in excess of 1e-10. Sequences with a database match were then further annotated using GO [52] (at 24/07/2007). Another view of the data was generated by Blast2GO [23] using the non-redundant (nr) database [53]. With the Blast2Go annotation, the programme GOSSIP [24] was run to identify any enrichment for GO annotations between the libraries. Sequence manipulation was carried out using the EMBOSS suite of programmes [54]. Sequences were clustered using ClustalW [55] and then subjected to phylogenetic analysis using the Phylip suite of programmes with bootstrapping [56] and displayed using MEGA4 [57]. Sequence alignments were displayed using BoxShade v3.21 [58].

## Authors' contributions

MC was the BAS Co-PI on the external funding, drafted the manuscript and lead the data analyses. MT performed the bioinformatic analyses. GG-L and JP assisted with animal collection and the physiological experiments to produce the cDNA libraries, JP also helped with the bioinformatic analyses. Michael Kube and Richard Reinhardt created the cDNA libraries and performed the sequencing of the clones. MRW was the BAS Co-PI on the external funding, performed the physiological experiments to produce the cDNA libraries and contributed to drafting the manuscript. All authors read and approved the final manuscript.
